# The Role of Electrochemical Immunosensors in Clinical Analysis

**DOI:** 10.3390/bios9030086

**Published:** 2019-07-09

**Authors:** Fariba Mollarasouli, Sevinc Kurbanoglu, Sibel A. Ozkan

**Affiliations:** 1 Department of Analytical Chemistry, Faculty of Pharmacy, Ankara University, 06560 Ankara, Turkey; 2 Department of Analytical Chemistry, Faculty of Chemistry, University of Tabriz, Tabriz 51666-16471, Iran

**Keywords:** clinical analysis, electrochemistry, immunosensors, biomarker, label free, labeled

## Abstract

An immunosensor is a kind of affinity biosensor based on interactions between an antigen and specific antigen immobilized on a transducer surface. Immunosensors possess high selectivity and sensitivity due to the specific binding between antibody and corresponding antigen, making them a suitable platform for several applications especially in the medical and bioanalysis fields. Electrochemical immunosensors rely on the measurements of an electrical signal recorded by an electrochemical transducer and can be classed as amperometric, potentiometric, conductometric, or impedimetric depending on the signal type. Among the immunosensors, electrochemical immunosensors have been more perfected due to their simplicity and, especially their ability to be portable, and for in situ or automated detection. This review addresses the potential of immunosensors destined for application in clinical analysis, especially cancer biomarker diagnosis. The emphasis is on the approaches used to fabricate electrochemical immunosensors. A general overview of recent applications of the developed electrochemical immunosensors in the clinical approach is described.

## 1. Introduction

### 1.1. What Is Immunosensor?

An immunosensor is a type of affinity solid-state based biosensor in which a specific target analyte, antigen (Ag), is detected by formation of a stable immunocomplex between antigen and antibody as a capture agent (Ab) resulting in generating a measurable signal given by a transducer [1,2]. There is a delicate distinction between immunosensors and immunoassays; an immunoassay system is based on the interaction between the antibody and the antigen in that the recognition process of the antigen takes place elsewhere [3,4]. However, in immunosensors, the formation of the immunocomplex and diagnosis take place on the same platform [5,6,7]. An example of the immunoassay system is the commercial Enzyme-Linked-Immunosorbent Assay (ELISA) used in the clinical assay and biochemical field [8]. In the conventional ELISA kits, a specific Ag is immobilized on a solid substrate and bonded to a specific Ab (primary Ab). In the last step, the Ab linked to an enzyme (as a type of label) is added and the antigen is sandwiched between the primary Ab and secondary Ab with an enzyme label. From this reaction, a detectable signal by changing color is readable by an optical transducer [9]. Since, ELISA is an optical immunoassay approach, it faces some drawbacks depending to the type of measurements such as direct ELISA, indirect ELISA, competitive ELISA, and sandwich ELISA. These limitations can be associated with the potential false signals arising from colored samples, a relatively long analysis time, a requirement of power-intensive light sources, monochromators and detectors, as well as sample size and usage problem outside the classical diagnostic laboratory [10]. In this regard, the use of immunosensors is a promising alternative to optical immunoassay approach for diagnosis of clinically important analytes due to high sensitivity and selectivity [5,11,12]. Furthermore, they provide the possibility of progression of immunoreactions at detector surfaces in real time. Immunosensors can be classified based on their transduction mode into three main class including optical (luminescence, fluorescence, refractive index), electrochemical (amperometric, potentiometric, impedance, and conductometric), and piezoelectric devices [9,13]. Among them, the use of electrochemical immunosensors simplifies the analysis with rapid and reliable signals. Electrochemical immunosensors are usually fabricated via the immobilization of a recognition element (i.e., antibody or antigen) on the electrode surface, which relies on measuring of currents and/or voltage resulting from binding between antibody and antigen [14,15,16,17,18,19]. Antibodies (Abs) are glycoproteins from the category of immunoglobulins (IgG, IgA, IgM, IgD, and IgE) made by specialized B lymphocyte cells of the host animal in response of the immune system to a foreign species called an antigen [20]. Among immunoglobulins, IgG, the most widely used in the developed immunosensors has “Y”-shaped molecules in which two identical light chains (molecular weights about 25,000 Da) and two heavy chains (MW about 50,000 Da) are linked together by disulfide bonds as well as noncovalent interactions (hydrogen bonds). Abs have physiological sites of action and variable regions for both chains, V_L_ and V_H_, depending on the amino acid sequences to bind the specific antigen. They are complementarity-determining regions (CDRs) providing hypervariable loops that indicate the binding site to the antigen. The high diversity of CDRs allows the production of a high specific antibody towards many kinds of Ag. Abs are bivalent and can bind with two specific Ags according to size, shape, and chemical compatibility. The Ag-binding site is called the “paratope”, and the complementary region on the Ag is called the “epitope”. The antibodies are classified into two types such as monoclonal antibodies (mAbs) and polyclonal antibodies (pAbs). Monoclonal antibodies are generated by identical immune B cells (clones of a single parent cell) and are used as a primary Ab in fabrication of immunosensors to recognize a single epitope of an antigen. On the other hand, the mAbs have monovalent affinity, resulting in high specificity towards an Ag. Polyclonal antibodies (pAbs) are usually produced by different immune B cell clones in the live animal body by injecting an immunogen. They are a heterogeneous mixture of immunoglobulins against a particular Ag which each of them can recognize and bind to different epitopes of a specific antigen. In contrast, mAbs are produced ex vivo by tissue-culture techniques [21].

**Figure 1:**
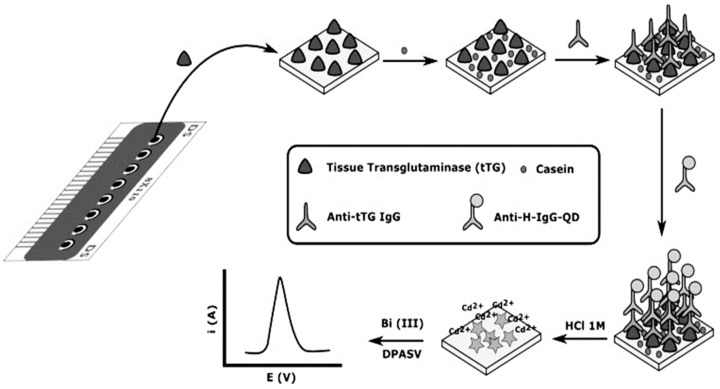
Schematic diagram of the electrochemical biosensor array. The bioassay is carried out using the working electrodes of the array as transducers and in situ electrochemical detection of QDs. Reprinted with Permission [22].

The aimed target in an immunosensor could be either an antibody (Ab) or an antigen (Ag). Although the most popular immunosensors are based on detecting antigen using antibodies, some researchers have reported an immunosensor for Ab detection. For example, in 2014 and 2015, Martín-Yerga et al. [22,23] described an electrochemical immunosensor for anti-tissue transglutaminase (anti-tTG) and anti-transglutaminase antibodies sensing, based on 8-channel screen-printed carbon electrodes (SPCE) modified with tissue-transglutaminase by adsorption. Transduction was performed by the immunoreaction with anti-human IgG labeled with CdSe/ZnS Quantum Dots (QDs) for the detection of Cd^2+^ released from QDs. The concentration of Cd^2+^ was measured in situ after acid attack of the QDs by voltammetric stripping without a transfer step (Figure 1). The electrochemical response was correlated with the anti-transglutaminase IgG concentration and the LOD of anti-tTG IgG antibodies was 2.2 U mL^−1^. Also in 2019, Gogola et al. [24] reported a label-free electrochemical immunosensor based on a gold electrode modified with Hantavirus (strain Araucaria) antigen/protein for detection of anti-Hantavirus antibody. The functionalization of the surface electrode was done by self-assembled monolayer (SAM) with 3-mercaptopropionic acid (MPA) diluted in acidified ethanol (99.6%) and then for the activation of the surface, *N*-(3-Dimethylaminopropyl)-*N*′-ethylcarbodiimide hydrochloride and N-Hydroxysuccinimide (EDC/NHS) were used. Therefore, the Hantavirus Araucaria nucleoprotein (HNp) could bind covalently onto the modified gold surface by immersing the electrode in the HNp solution. Finally, the Au-MPA-HNp electrode was immersed in the solution containing the antibody to couple the antigen with antibody. Transduction was made by using electrochemical impedance spectroscopy (EIS) measurements. The detection range was from 0.400 to 300 μg mL^−1^.

Detection approaches are divided into label-free and labeled assays. Label-free assays are performed by measuring the presence of an analyte directly via biochemical reactions on a transducer surface, while in the labeled assays, the analyte is sandwiched between the capture agent and labeled agent with a special label such as an enzyme, quantum dot, fluorophore, or radioisotope, for obtaining signal.

#### 1.1.1. Direct Immunosensors

Direct immunosensors, in other words label free immunosensors, are capable of detecting the physical or chemical changes arising directly from the immune antigen-antibody complex formation without needing to label. In this type of immunosensor, there is no need to use the label and it can be applied for fast, real-time analysis. Hence, the application of label free immunosensors in a clinical approach has become increasingly favoritized in the last decades. However, the label free immunosensors suffer from a significant effect of non-specific adsorption on their response [11,25,26,27]. Generally, no signal should be observed in the absence of an antigen–antibody (Ag–Ab) interaction, but a small signal is always obtained due to the non-specific binding of the antigen or others proteins on the surface of the substrate. Non-specific adsorption can occur when the other proteins existing in the sample, which can adsorb to antibodies or support surface, lead to an increase in the background signal. This phenomenon results in decreasing sensitivity. Hence, the use of a proper blocking agent is necessary. Several compounds are used as blocking agents such as surfactants (Tween 20, polyethylene glycol), casein, bovine serum albumin (BSA), and thionic compounds for gold surfaces [28,29,30,31].

#### 1.1.2. Indirect Immunosensors

Indirect immunosensors, generally labeled immunosensors, are based on the signal generating from one or several labels allowing highly sensitive and versatile detection. In indirect measurements such as enzymes, e.g., horseradish peroxidase [32], catalase and glucose oxidase [33], alkaline phosphatase [34], and electroactive compounds as a mediator such as ferrocene, Prussian blue is usually used in the electrochemical immunosensor, especially when antigens and antibodies are not intrinsically electroactive. Moreover, several nanomaterials such as gold nanoparticles (Au NPs) or different quantum dots (QDs) can be used to amplify the signal. In the labeled immunosensors, labels can be attached to an antibody or antigen to achieve an electron-transfer and assuming that the number of labels detected during measurement correspond to the number of target analytes. In contrast to label free, labeled immunosensors have some advantages such as higher sensitivity and a lower effect of non-specific adsorption on the signal, but face some drawbacks like; the effect on antigen antibody binding efficiency on labelling a biomolecule due to the variability of the biomolecule label coupling reaction [19,28,30,35].

Labeled immunosensors can be divided into two other types of possible formats: competitive format and non-competitive format.

##### Competitive Immunosensors

Competitive immunosensors are useful for small antigens with one epitope in which the sample analyte and labeled analyte compete with each other to access the limited number of antibody-binding sites. Due to the use of the limited amount of antibody, this format is known as “limited reagent assay”. In general, antibody is firstly immobilized on the substrate surface and then incubated with a mixed solution of a known amount of labeled antigen and an unknown sample. The signal obtained from the labeled analyte is inversely proportional to the sample analyte amount. Thus, a larger signal is obtained for a fixed number of antibody sites when the quantity ratio of sample to labeled analyte is low [19,28,30,35].

**Figure 2:**
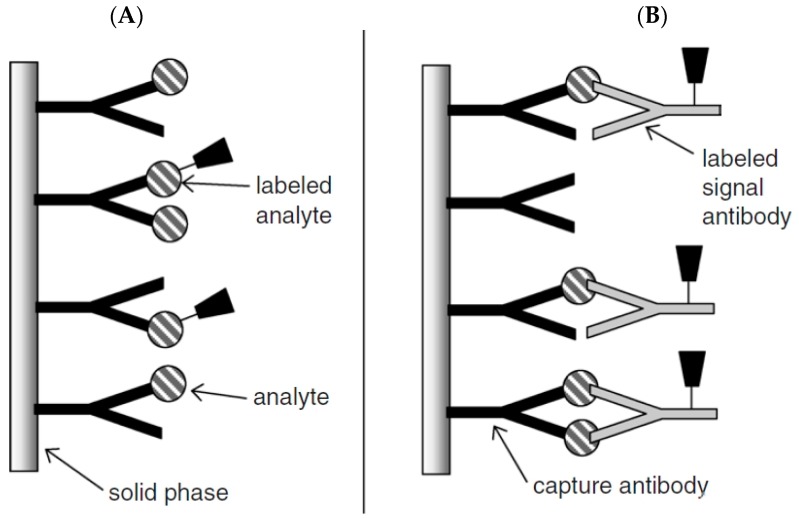
Schematic representation of (**A**) competitive and (**B**) non-competitive immunoassay formats. Reprinted with Permission [35].

Competitive immunoassays can be carried out in two different ways e.g., homogenous or heterogeneous approach. In the homogenous assay, the amount of labeled unbound antigen is measured with no need for separation steps, but in the heterogeneous assay, the labeled bound antigen is measured after removing the labeled unbound antigen by using a washing step. Although the homogenous assay is faster than the heterogeneous, the sensitivity is lower than that due to non-specific adsorption. The scheme principle of competitive immunosensors is given in Figure 2A [35] There are several papers based on competitive immunosensors [36,37].

**Figure 3:**
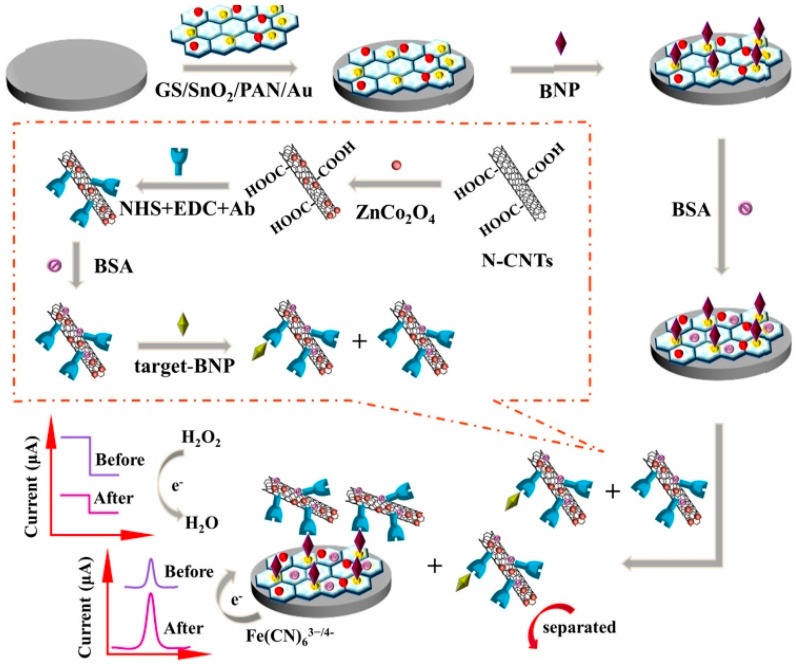
The schematic illustration of the dual mode competitive electrochemical immunosensor. Reprinted with Permission [38].

In 2019, Li et al. described a dual competitive electrochemical immunosensor based on differential pulse voltammetry (DPV) and amperometric i-t curve response modes for the detection of B-type natriuretic peptide (BNP) [38]. In this work, glassy carbon electrode was modified with GS/SnO2/PAN-Au which acted as the platform for the DPV signal and ZnCo_2_O_4_/N-CNTs complex acted as the label for providing an amperometric signal for the reduction of H_2_O_2_ (Figure 3). Under the optimum conditions, the immunosensor displayed a remarkable analytical performance with a linear range from 0.01 pg/mL to 1 ng/mL and a detection limit of 3.4 fg/mL for the determination of BNP (S/N = 3). This method has been suggested to become a universal approach for other biological detection.

A competitive immunosensor was also developed by Zhang et al. in 2019, for ultrasensitive detection of microcystin-LR (MC-LR) using metal-organic frameworks (MOFs) material [39]. Anti-MC-LR was immobilized on the glassy carbon electrode surface modified by electrodeposition of graphene oxide (GO). Au NPs@MIL-101 was used as a label to detect MC-LR using the competitive method and had a catalytic effect on the oxidation of ascorbic acid (AA) to achieve MC-LR detection. With optimizing of the conditions, the sensitivity and selectivity of the MC-LR immunosensors are significantly improved with a wide linear range from 0.05 ng/mL to 75 μg/mL and a low detection limit of 0.02 ng/mL (S/N = 3). For detection of cortisol in human saliva, Kämäräinen et al. described an electrochemical immunosensor based on a direct competitive enzyme linked immunoassay using a disposable graphite screen-printed electrode. The electroanalytical response was generated by using an alkaline phosphatase (AP)-conjugated cortisol and 1-naphtyl phosphate as enzyme substrate. Square wave voltammetry (SWV) was used to monitor the enzyme activity. A low LOD of 0.6 ng/mL was obtained [40].

##### Non-Competitive Immunosensors

Non-competitive immunosensors, known as two-site “sandwich” immunoassays, are applied for large antigens with epitopes of more than one. In this assay, excess amounts of secondary and primary antibodies are used and the antigen is sandwiched between two antibodies. An antibody which is immobilized on the solid substrate surface is called a capture antibody (primary) that captures the antigen from the sample and another antibody is labeled antibody (secondary) which binds to the antigen–antibody complex, leading to production of a detected signal (Figure 2B). The signals usually arise from catalytic reaction of the enzyme labeled as a probe with the detection antibody. A washing step is necessary after each incubation step to remove unbound reagents [19,28,30,35].

A “one-site” assay is based on the immunoreaction between an immobilized antigen and a labeled antibody. The unbound labeled antibody is removed by washing to prevent precipitation in the binding event, so that the signal obtained by the bound antibody can be measured. Although the non-competitive sandwich format, as a common format of immunosensors, has higher sensitivity, specificity, and a wider dynamic range in compression to the competitive assays, it is not usable for the assay of small molecules (monovalent with molecular weight < 1000 Da) [19,28,30,35].

### 1.2. Electrochemical Immunosensing

The electrochemical immunosensors can be also classified according to the applied technique in potentiometric, conductometric, impedance, and amperometric mode [3,12,19,28,30,35,41,42,43].

#### 1.2.1. Amperometric Immunosensors

Most electrochemical immunosensors are based on amperometric measurement by applying a constant potential at the working electrode related to the reference electrode in which a current is obtained from the electrochemical oxidation or electroreduction of an electroactive species. Only a small number of label free immunosensors have been reported as amperometric immunosensors [44] because most antibodies and antigens are not electroactive and cannot give amperometric response. Hence, in the majority of amperometric immunosensors, the use of electroactive labels or mediators is required to achieve a response for the detection of biomarkers. The major disadvantage of the amperometric immunosensor is limitation to the indirect detection system, however, it is compensated for by excellent sensitivity due to a linear relationship between the immunosensor response and the concentration of analyte (antigen), compared to a logarithmic relationship for potentiometric systems [9,30].

#### 1.2.2. Potentiometric Immunosensors

Potentiometric immunosensors are based on measuring the change of potential due to the formation of immunocomplex between antibody and antigen. The sensitivity of this type is lower than others because the difference of potential is small during the immunoaffinity reaction as well as the effect of interferences being serious. Some of the label-free immunosensors belong to this group [45]. The main advantage of potentiometric immunosensors is the simplicity of operation, allowing use in automation and miniaturization of solid-state sensors [30,35].

#### 1.2.3. Impedimetric Immunosensors

Electrochemical impedance spectroscopy (EIS) is an important and useful technique widely used for surface characterization in chemical sensors and biosensors especially for label-free detection. In addition, it can be used for the investigation of the electrode/electrolyte interface and for study of the kinetics of the electrode surface and the binding kinetics between molecules such as receptors, DNAs, proteins, antibodies, antigens, ions etc. The fundamental effect of EIS is based on applying a constant AC potential with a small perturbation, usually 5 or 10 mV, over a wide range of frequencies. The results are then plotted in a Nyquist plot where the imaginary part of impedance (−Z’’) is plotted versus the real part of impedance (Z’) and/or in a Bode plot, where the total impedance (|Z|) is plotted versus the frequency [46].

Most of the impedimetric model circuits (Randless cell circuit) designed are based on elements of electrolyte solution resistance (Rs), Warburg impedance (W), charge transfer resistance (R_ct_), and a pure capacitor (C). EIS is widely used for the development of label-free immunosensors. This type of immunosensor is very suitable for the detection of various target biomarkers, resulting in monitoring the formation of antibody-antigen directly without the need of a further label. The response is based on the change of interfacial properties (charge–transfer resistance) related to the immune-recognition event [40,41,42,43]. The advantages of this type of immunosensor are simple and fast detection, low cost, insensitivity to environmental condition changes, no sample pretreatment, and the application for the detection of a wide range of analytes. For label-free detection of carcinoembryonic antigen (CEA) by using the impedimetric method, Ganganboina and Doong reported an immunosensor based on a Pt electrode modified by casting of N, S-graphene quantum dots@Au-polyaniline (N, S-GQDs@Au-PANI) nanowires on the electrode surface [47]. Using Fe(CN)_6_^3−/4−^ as redox probe in solution and EIS as electrochemical technique, the immunosensor was sensitive to the concentration of the CEA on the immobilized Ab, showing an LOD of 0.01 ng mL^−1^ and a linear detection range between 0.5 and 1000 ng mL^−1^. The N, S-GQDs@Au-PANI nanowires as excellent conducting materials can accelerate the electron transfer, but after the formation of CEA antibody-antigen bio conjugates by adding CEA, the charge transfer resistance is significantly increased, and subsequently a highly stable and label-free immunosensor platform is fabricated for the impedimetric detection of CEA.

#### 1.2.4. Conductometric Immunosensors

Conductometric immunosensors rely on a relationship between conductance and a bio-recognition event. When the reaction occurs between a bio-recognition element and an antigen, the conductivity of the solution or current flow is changed due to the change in ionic species concentration. On the other hand, the conductivity of the supporting electrolyte is changed when antibodies labeled with enzyme are conjugated with antigens in the sample solution, the enzyme’s performance stops by the inhibitory effect of the antigen–antibody complex blocking the surface of the electrode. This signal can be measured by an ohmmeter or multimeter. Conductometric immunosensors have a number of advantages, including low driving voltage, large-scale production, and suitability for miniaturization without a reference electrode [48].

### 1.3. Immobilization Methods

Since the immobilization procedure of antibody on the electrode surface plays a critical role in the signal obtained by the specific immunoreactions between an immobilized antibody and antigen, the choice of an appropriate immobilization approach is a major step for achieving a high performance immunosensor. In addition, the proper immobilization method for the antibody can guarantee the stability and applicability of the affinity biosensor. For this purpose, a more appropriate method is that, first, it can maintain the biological activity of the bioelement (antibody or aptamer) and second, it provides a good orientation in terms of binding sites exposed towards the aimed analyte as well as a proper density [30]. A high density of the bioelement can hinder the binding of antigen. Hence, the bioelement layer needs to be immobilized on the surface of the sensor by various methods [36,49,50].

#### 1.3.1. Physical Adsorption

Physical adsorption is the simplest method for binding an antibody to the electrode surface based on non-covalent interactions between the bioelement and the surface of the sensing device, including electrostatic interactions, hydrogen bonding, van der Waals, and hydrophobic interaction. The immobilization of antibodies on the ELISA micro-titer plate or on the self-assembled monolayer (SAM) of gold nanoparticles with thiol groups, via electrostatic adsorption attractions, are common examples for the adsorption method. If the concentration of antibody is sufficient, a layer of proteins is formed on the electrode surface [49]. The application of a physical adsorption strategy faces some limitations due to the problems arising when the concentration of the immobilized antibody is very low forming dense packing. Many factors may affect the quality of immunosensors based on physical adsorption such as (i) surface contamination at a low concentration of antibody; (ii) blocking the idiotypic sites of IgG when the adsorption takes place near to the substrate surface, leading to loss of binding capacity; (iii) partial denaturation of IgG [49]. Direct physical adsorption of bioelements on solid substrata has a random orientation of bioelements and a probability of desorption due to weak bonds resulting in low sensitivity and reproducibility [18], conformational changes, as well as decreased bioactivities with time [49,51,52,53,54]. Despite these drawbacks of antibody adsorption, these methods are used in many applications such as antibody arrays, ELISA, and immunosensors, due to their simple and easy procedure, and high antibody-binding capacity [55].

#### 1.3.2. Trapping Methods

This procedure is based on the encapsulation of bioelements in polymer matrices such as molecularly imprinted polymers (MIP) and sol-gel. The trapping method possesses more stability in comparison to the physical adsorption procedure. The sol-gel-based immobilization method has a number of advantages such as simplicity, low-temperature encapsulation of bioelements, optical transparency, high thermal stability, tunable porosity, mechanical rigidity, and low chemical reactivity [49,52,53,54,56].

#### 1.3.3. Covalent Binding

The covalent binding strategy as the most common method is used in the immobilization of bioelements based on covalent interactions between the surface of the modified electrode and the functional groups of antibodies or aptamers [18]. Functional groups of antibodies for covalent binding can include amine, carboxyl, carbohydrate, and thiol moieties. The covalent attachment depends on the various chemistries such as substrate functionality, functional groups on the antibody, and some physical conditions like pH, temperature, and degree of conjugation [57]. Generally, electrode surfaces have no sites for covalent bonding, so it is necessary to first coat a thin film of the functional groups in order to covalently bond with the amino groups of the antibodies for example. For this purpose, some reagents such as glutaraldehyde, carbodiimide succinimide ester, N-hydroxysuccinimide, periodate, or maleinimide can be used. Glutaraldehyde is suitable for smaller sized molecules and N, N′-carbonyldiimidazole is recommended for higher sized molecules (>20 nm). Glutaraldehyde has two aldehyde groups and can react as a bi-functional cross-linking agent with amine groups to form peptide bonds [58,59]. Another approach to obtain functionalized films on the surface electrode for the immobilization of bioelements (e.g., antibodies, antigens and aptamers) is the self-assembled monolayer (SAM) technique [60,61]. Based on this strategy, semi-covalent binding is formed between a sulfide group (disulfides, sulfides and thiols) and the noble metal surfaces (e.g., Au, Ag or Pt) to form highly organized SAMs. Thiol functionalized antibodies can be easily immobilized on Au surfaces [62]. The formation of thin layers is perfected because it reduces the diffusion time of the analytes and improves the speed of the sensor response. In addition, because most of the reactants used in these systems are organic molecules, a thinner layer will have a lower insulating effect. The use of oligo-ethylene glycol thiol owning to its regular structure, high biocompatibility, minimal desorption as well as the functionalized alkyl silanes with amine, aldehyde, thiol, or carboxylic groups is another common strategy for cross-linking [58]. The covalent attachment method of proteins has been reported in much detail in some reviews [57,63]. This procedure is more stable and has better oriented immobilization [64,65].

#### 1.3.4. Affinity Immobilization Techniques

Affinity immobilization techniques are favorable strategies for obtaining high capture efficiency and oriented immobilization of the biorecognition elements to achieve a high performance immunosensor. Bio-affinity immobilization techniques are based on material binding peptides, Protein A or G, biotin–streptavidin interaction, Fc-binding peptides and aptamers, lectin-sugar, His- Tag systems, nucleotide binding site, and DNA-directed immobilization [54,66,67,68].

Protein A or G are small bacterial proteins which specifically bind to the Fc regions of antibodies, allowing oriented immobilization to be obtained on the solid support. Protein A was originally isolated from Staphylococcus aureus and shows five Fc binding domains placed near the terminal-NH_2_ [69].

(Strept)avidin-biotin is widely used for oriented immobilization of various biomolecules such as polysaccharides, nucleic acids, and protein like antibodies in which biotinylated capture antibodies are bound to avidin or streptavidin surfaces. Biotin–(strept)avidin has a strong non-covalent interaction which is resistant at high temperatures, pH variations as well as exposure to chemicals (e.g., protein denaturants) [70,71,72,73,74].

## 2. Recent Applications of Electrochemical Immunosensors in Clinical Analyses

**Figure 4:**
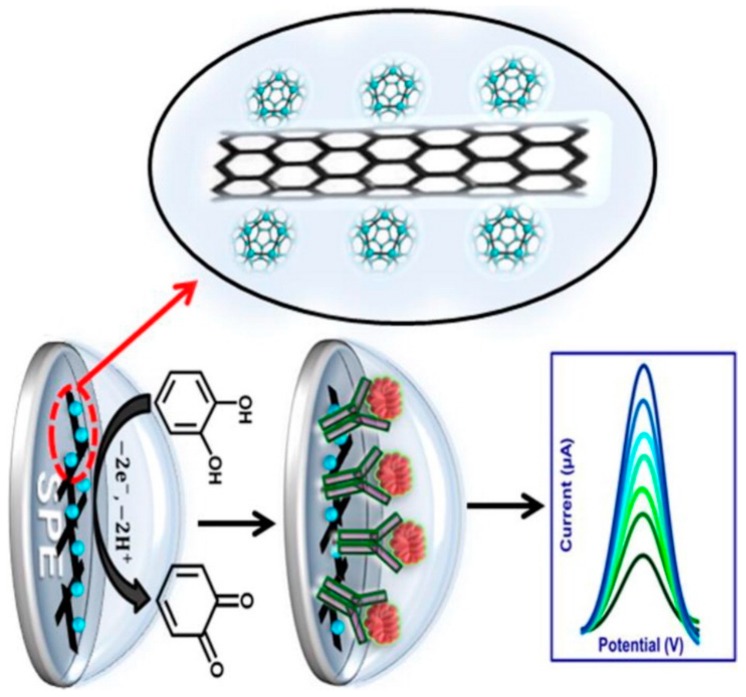
Schematic representation of Fullerene-Functionalized Carbon Nanotubes/Ionic Liquid based label-free electrochemical immunosensor. Reprinted with Permission [75].

Immunosensors can be designed for cancer biomarkers, autoimmune diseases, cardiac diseases, etc. In their study, Mazloum-Ardakani et al., developed a Label-Free Electrochemical Immunosensor for Detection of Tumor Necrosis Factor α Based on Fullerene-Functionalized Carbon Nanotubes/Ionic Liquid (C_60_–CNTs–IL) (Figure 4). The biosensor has a linear range between 5.0 pg·mL^−1^ and 75 pg·mL^−1^ with a low detection limit of 2.0 pg·mL^−1^ for TNF-α. Moreover, the designed label-free electrochemical immunosensor was successfully applied to serum samples [75].

AFP, which is a liver, ovarian, testicular cancer biomarker, was detected using one-step electrochemical immunoassay by Chen and co-workers. Horseradish peroxidase–anti-AFP (HRP–anti-AFP) was immobilized on a nanogold-functionalized graphene interface and antigen–antibody complex formation was followed by HRP for the catalytic reduction of H_2_O_2_ in the solution. AFP was detected in the linear range of 0.1–200 ng·mL^−1^ with a detection limit (LOD) of 0.05 ng·mL^−1^ AFP [76].

Carcino-embryonic antigen (CEA) was detected using a novel label-free electrochemical immunosensor based on PtPd bimetallic nanoparticles graphene quantum dots and gold nanoparticles (PtPd/N-GQDs@Au). Due to the large surface area, high conductivity, and high biocompatibility, the biosensor showeda linear range between 5 fg/mL and 50 ng/mL with a very low detection limit of 2 fg/mL which is of the order 10^−15^ [77]. In another work, simultaneous detection of CEA and AFP was suggested using gold nanoparticles decorated multiwall carbon nanotubes. A novel sandwich-type electrochemical immunosensor was developed with a linear range of 0.01 to 60 ng mL^−1^ for CEA and AFP and with detection limits of 3.0 pg mL^−1^ for CEA and 4.5 pg mL^−1^ for AFP [78]. In their work, Stoeva et al., developed a biosensor for multiple detection of protein cancer biomarkers; CEA, CA 125, CA 153, CA 199. A screen printed array was used for this purpose, and linear ranges of 0.16–9.2 ng/mL for CEA, 0.084–16 U/mL for CA 153, 0.11–13 U/mL CA 125, 0.16–15 U/mL for CA 199 were obtained with LOD values of 0.04 ng/mL CEA, 0.06 U/mL CA 153, 0.03 U/mL CA 125, 0.1 U/mL CA 199 [79].

**Figure 5:**
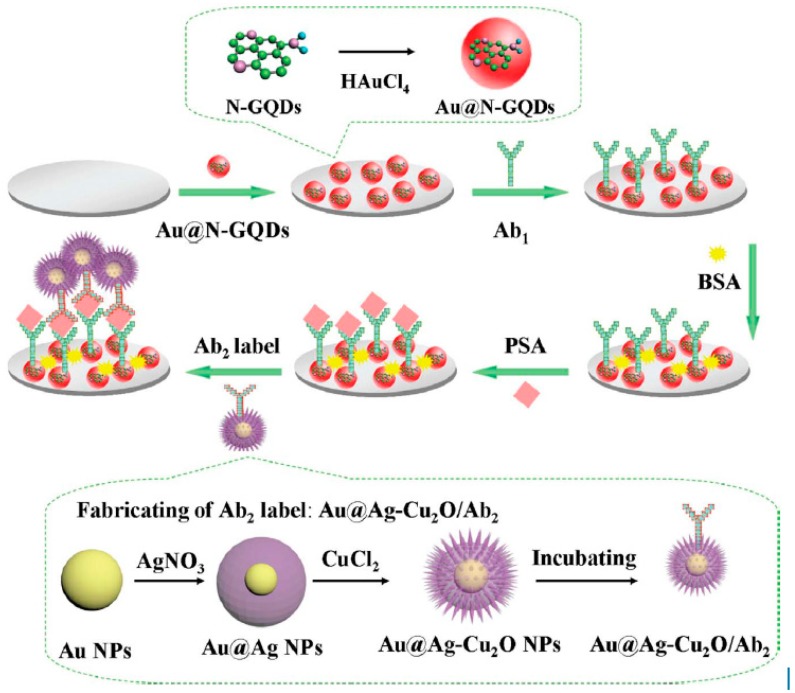
The schematic illustration of the sandwich-type electrochemical immunosensor and the preparation procedure of Au@N-GQDs NPs and Au@Ag-Cu_2_O/Ab_2_ Reprinted with Permission [82].

IL-6, which is rheumatoid arthritis, systemic lupus erythematosus biomarker, was detected using Poly-HRP labeled anti-IL-6 antibodies, Gold compact disc, and an 8-electrode array system. IL-6 detection was obtained within a linear range of 10–1300 fg/mL with an LOD value of 10 fg/mL [80]. In another work, a 16-electrode SPCE array was used for ultrasensitive multiplexing electrochemical immunosensing of PSA with IL-8. LOD values of 5 pg/mL and 8 pg/mL, for PSA and IL-8, respectively [81]. An ultrasensitive sandwich-type electrochemical immunosensor was developed by Yang et al., using gold nanoparticles functionalized nitrogen-doped graphene quantum dots (Au@N-GQDs) for Prostate specific antigen (PSA). Highly specific interaction between antigen and antibody was obtained with a dynamic range from 0.01 pg.m^−1^ to 100 ng·mL^−1^ with a low detection limit of 0.003 pg·mL^−1^ followed by the reduction of hydrogen peroxide (H_2_O_2_) with amperometric response at −0.4 V [82]. (Figure 5).

Herein, we report the research progress of electrochemical immunosensors applied in clinical analysis that were published in the last years (Table 1).

## 3. Conclusions

Every protein is a biomarker of one or more diseases. CA 15-3, CA 27-29, HER2/NEU are biomarkers for Breast cancer, CA 125 Ovarian CEA Colon is a biomarker of cancer, PSA is a biomarker of the Prostate, NMP22, Fibrin/FDP, BTA, high molecular weight CEA are biomarkers for Bladder cancer, α-fetoprotein and Human gonadotropin-β are biomarkers for Testicular cancer, CA 19-9 is a biomarker of Pancreatic cancer. The level of determination of these biomarkers is important to see if they exist or not in the sample with rapid and reliable detection. Cut-off values of these biomarkers gives information about whether the patient is ill or not, and gives the analytical chemist the opportunity to develop methods that can detect these or even lower values accurately and precisely. All the biomarkers have unique and different cut off values such as, TNF-α has cut-off value of 11.63 pg·mL^−1^ whereas CRP has a cut-off value of 60 mg·L^−1^. The analytical chemist should discuss these cut-off values and find suitable analytical methods for immunosensing. Electrochemical methods especially impedance based ones give a ground for choice by researchers and companies as they give a rapid response and a specific electrochemical response towards analyte. Electrochemical immunosensors in clinical analyses have frequently found a place due to their rapid response in terms of “exist or not exist”. We believe that as the technology is developing very quickly, there will be picomolar level sensors as early grade detection of illness, save lives.

## Figures and Tables

**Table 1:** Some selected studies on electrochemical immunosensors in clinical analyses.

Biomarker	Type of Illness	Immunosensor		Electrochemical Technique	Linear Range	LOD	Reference
AFP	Liver, Ovarian, Testicular Cancers	Nanogold/TH-*f* GRNS-AuE	Amperometry	0.1–200 ng·mL^−1^	0.05 ng·mL^−1^	[76]	
AFP	Liver, Ovarian, Testicular cancers	AuNPs/graphene-doped CS/TH-GCE	Amperometry	1–10 ng·mL^−1^	0.7 ng·mL^−1^	[83]	
AFP	Liver, Ovarian, Testicular Cancers	GRS/CS-CE	Square wave voltammetry	0.05–6 ng·mL^−1^	0.02 ng·mL^−1^	[84]	
AXL	Prostate	anti-AXL/GQDs/SPCE	Differential pulse voltammetry	1.7–1000 pg·mL^−1^	0.5 pg·mL^−1^	[45]	
CEA	Breast Colorectal and Pancreatic, Liver, Lung, Ovarian, Colon, Bladder Cancers	GOx/HRP MWCNT anti/CEA TH/AuNPs-decorated dendrimer-cysteamine/AuE	Square wave voltammetry	10 pg·mL^−1^–50 ng·mL^−1^	4.4 ± 0.1 pg·mL^−1^	[85]	
CEA	Breast Colorectal and Pancreatic, Liver, Lung, Ovarian, Colon, Bladder Cancers	Nitrogen-doped GQDs/Pt-PdBiMNP AuNPs/GCE	Amperometry	5 fg·mL^−1^–50 ng·mL^−1^	2 fg·mL^−1^	[77]	
CEA	Breast Colorectal and Pancreatic, Liver, Lung, Ovarian, Colon, Bladder Cancers	Trimetallic NiAuPt NPs(GRNS/β–cyclodextrin/GONS/GCE	Amperometry	0.001–100 ng·mL^−1^	0.27 pg·mL^−1^	[86]	
CEA	Breast Colorectal and Pancreatic, Liver, Lung, Ovarian, Colon, Bladder Cancers	AgNPs/MWCNTs/MnO_2_ labeled anti-CEA antibodies β–cyclodextrin/MWCNT/GCE	Amperometry	NS	0.03 pg·mL^−1^	[87]	
CEA	Breast Colorectal and Pancreatic, Liver, Lung, Ovarian, Colon, Bladder Cancers	Bismuth film/GCE	Square wave voltammetry	0.05–25 ng·mL^−1^	5 pg·mL^−1^	[88]	
CEA	Breast Colorectal and Pancreatic, Liver, Lung, Ovarian, Colon, Bladder Cancers	AuNPs/poly(styrene-co-acrylic acid) microbead labeled anti-CEA antibodies CS/graphene oxide film/GCE	Linear sweep voltammetry	0.5 pg·mL^−1^–0.5 ng·mL^−1^	0.12 pg·mL^−1^	[89]	
CEA AFP	Breast Colorectal and Pancreatic, Liver, Lung, Ovarian, Colon, Bladder Cancers Liver, Ovarian, Testicular Cancers	Cd (II)/Au NPs@MWCNTs labeled anti-CEA antibodies Pb (II)/Au NPs@MWCNTs labeled anti-AFP antibodies AuNPs/AuE	Square wave voltammetry	0.01–60 ng·mL^−1^	3 pg·mL^−1^ CEA 4.5 pg·mL^−1^ AFP	[78]	
CEA AFP	Breast Colorectal and Pancreatic, Liver, Lung, Ovarian, Colon, Bladder Cancers Liver, Ovarian, Testicular Cancers	PtPNPs/Cd(II) labeled anti-CEA antibodies PtPNPs/Cu(II) labeled anti-AFP antibodies Graphene oxide/GCE	Amperometry	0.05–200 ng·mL^−1^ 0.05–200 ng·mL^−1^	0.002 ng·mL^−1^ CEA 0.05 ng·mL^−1^ AFP	[90]	
CAS	Bladder Cancer	Bismuth film-modified nylon membrane-foldable SPCE	Square wave voltammetry	0–5 µg·mL^−1^ CAS	0.04 µg·mL^−1^ CAS	[91]	
CEA CA 125 CA 153 CA 199	Breast Colorectal and Pancreatic, Liver, Lung, Ovarian, Colon, Bladder Cancers Breast Cancer	4-electrode SPCE array	Differential pulse voltammetry	0.16–9.2 ng·mL CEA 0.084–16 U·mL^−1^ CA 153 0.11–13 U·mL^−1^ CA 125 0.16–15 U·mL^−1^ CA 199	0.04 ng·mL^−1^ CEA 0.06 U·mL^−1^ CA 153 0.03 U·mL^−1^ CA 125 0.1 U·mL^−1^ CA 199	[79]	
HER2-ECD CA 15-3	Breast Cancer	SPCE-AuNPs	Square wave voltammetry	0–50 ng·mL^−1^ (HER2-ECD) 0–70 U·mL^−1^ (CA 15-3)	2.9 ng·mL^−1^ (HER2-ECD) 5.0 U·mL^−1^; (CA 15-3)	[92]	
IL-6	Rheumatoid Arthritis Systemic Lupus Erythematosus	Poly-HRP labeled anti-IL-6 antibodies Gold compact disc 8-electrode array	Amperometry	10–1300 fg·mL^−1^	10 fg·mL^−1^	[80]	
IL-6	Rheumatoid Arthritis Systemic Lupus Erythematosus	HRP labeled anti-IL-6 antibodies AuNPs-modified 8-electrode array	Amperometry	20–400 pg·mL^−1^	20 pg·mL^−1^	[93]	
IL-6	Rheumatoid arthritis Systemic lupus erythematosus	HRP/MWCNT labeled anti-IL-6 antibodies Single-wall-PGDE	Amperometry	NS	0.5 pg·mL^−1^	[94]	
PSA	Prostate	Silver hybridized mesoporous silica nanoparticles Signal amplifier-modified GCE	Amperometry	0.15–20 ng·mL^−1^	0.06 ng·mL^−1^	[95]	
PSA	Prostate	Multi-HRP/MWCNT labeled anti-PSA antibodies SWCN-CE	Amperometry	NS		[96]	
PSA	Prostate	AuNPs/PAMAM/HRP labeled PSA aptamer Graphene oxide/CS/TH film-modified GCE	Electrochemical impedance spectroscopy	5 pg·mL^−1^–35 ng·mL^−1^	5 pg·mL^−1^	[97]	
PSA	Prostate	Au/Ag-graphene oxide/GQDs labeled anti-PSA antibodies Signal amplifier-modified GCE	Electrochemiluminescent	1 pg·mL^−1^–10 ng·mL^−1^	0.29 pg·mL^−1^	[25]	
PSA	Prostate	HRP-modified magnetic particles labeled anti-PSA antibodies AuNPs-modified pyrolytic graphite disk electrode	Amperometry	4–10 ng·mL^−1^	0.5 pg·mL^−1^	[98]	
PSA	Prostate	Anti-PSA/MWCNTs/IL/GCE	Differential pulse voltammetry	0.2–1.0 ng·mL^−1^ 1-40 ng·mL^−1^	20 pg·mL^−1^	[50]	
PSA IL-6	Prostate Rheumatoid Arthritis Systemic Lupus Erythematosus	AuNPs-microfluidic 8-electrode SPCE array	Amperometry	0.23 pg·mL^−1^ PSA 0.30 pg·mL^−1^ IL-6		[99]	
PSA hCG	Prostate Cancer Ovarian, Testicular, Trophoblastic Cancers	Porous membrane-coated 2-electrode gold array	Amperometry	NS	0.4 µg L^−1^ PSA 2.5 U L^−1^ hCG	[100]	
PSA IL-8	Prostate Rheumatoid Arthritis, Inflammatory Bowel Disease, Psoriasis, Acute Respiratory Distress Syndrome	16-electrode SPCE array	Amperometry	-	5 pg·mL^−1^ PSA 8 pg·mL^−1^ IL-8	[81]	
PthA	Citrus Bacterial Cancer Disease	AuNP/PB/CILE/GCE	Square wave voltammetry	0.03–100 nM	0.01 nM	[101]	
TNF-α	Rheumatoid Arthritis	PA+PAA/GCE	Amperometry	0.02–200.00 ng·mL^−1^	0.01 ng·mL^−1^	[34]	
TNF-α	Rheumatoid Arthritis	K3[Fe(CN)6]-CHT/GA/NA/mouse anti-human TNF-α	Cyclic voltammetry	0.02–34 ng·mL^−1^	10 pg·mL^−1^	[43]	
TNF-α	Rheumatoid Arthritis	C60-fMWCNT-IL	Differential pulse voltammetry	5.0–75 pg·mL^−1^	2.0 pg·mL^−1^	[75]	
TNF-α	Rheumatoid Arthritis	Microfluidic	Differential pulse voltammetry	3.25–50 ng·mL^−1^	4.1 ng·mL^−1^	[102]	
TNF-α	Rheumatoid Arthritis	Dibutyl phthalate/polyvinyl chloride matrix	Potentiometry	0.1–1.0 mg·L^−1^	0.015 mg·L^−1^	[103]	

## Abbreviations

TNF-α: tumor necrosis factor-alpha; PB: protein biomarker; anti-CCP: anti-cyclic citrullinated peptide; IL: interleukin; ACAB: anti-chromatin (anti-nucleosome) autoantibodies; CRP: C-reactive protein; TA-Ab: transglutaminase autoantibody; IL-17RA: interleukin-17 receptor A; MBP: myelin basic protein; IBD: inflammatory bowel disease; PS: psoriasis; ARDS: acute respiratory distress syndrome; MS: multiple sclerosis; n.s.: not stated. CA: cancer/carbohydrate antigen; CEA: carcinoembryonic antigen; HER2/NEU: human epidermal growth factor receptor 2; PSA: prostate specific antigen; NMP22: nuclear matrix protein 22; FDP: fibrin degradation product; BTA: bladder tumor associated antigen. PA: Polyaniline; PAA: polyacrylic acid; CHT: Chitosan; GA: glutaraldehyde; NA: nafion; GRNS: graphene nanosheets; Pt–PdBiMNP: platinum–palladium bimetallic nanoparticles; AuNPs: gold nanoparticles; CE: Carbon electrode; GONS: Graphene oxide nanosheets PGDE: pyrolytic graphite disk electrode C_60_: fullerene.
